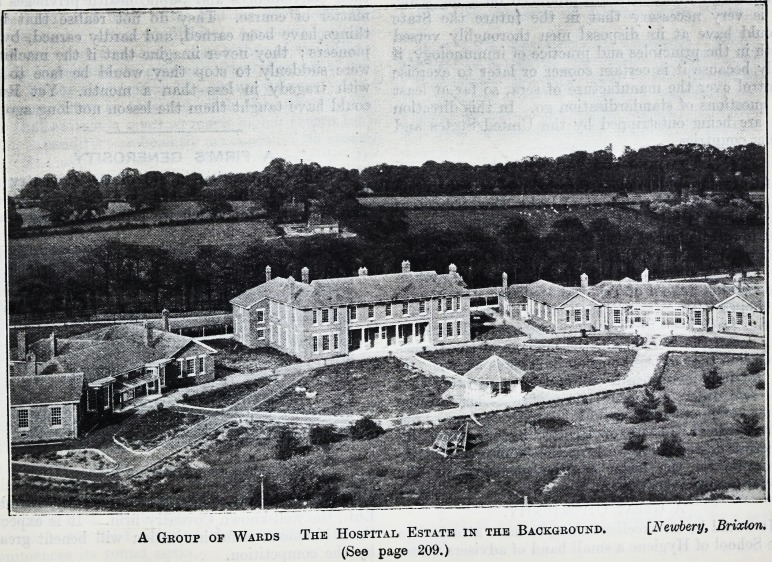# West Park Mental Hospital, Epsom

**Published:** 1924-07

**Authors:** 


### WEST PARK MENTAL HOSPITAL.

Friday, June 20, was an outstanding day in the history of the London County Council's achievements, for it was the opening day of their fine new mental hospital at West Park, Epsom. At a ceremony at which the hospital was opened by the Minister of Health, the chair was taken by Mr. J. Herbert Hunter, chairman of the Council, and particulars of the hospital were given by the Hon. Wm. Sidney, chairman of the Mental Hospitals Committee. During the ceremony stress was laid upon the fact that the Council and officers of the hospital wish it to be regarded, not as the old-fashioned asylum was regarded, but as a hospital where sick minds are tended. The hospital, designed by Mr. W. C. Clifford Smith, O.B.E., F.R.I.B.A., Mental Hospitals Engineer to the London Council Council, stands in beautiful grounds of 200 acres and will accommodate over 2,000 patients with a staff of about 300. It is built on the villa principle, every building, of which there are 28, being detached. A remarkable feature is the open villas for patients engaged in domestic duties and work on the farm and in the gardens, who will be under no restrictions with regard to remaining in the hospital grounds other than their own word of honour. Every form of mental deficiency or breakdown will be treated, and there are special wards for epileptics and special hospitals for the treatment of tuberculosis, dysentery, and other infectious diseases. When it is completed the cost of the hospital is estimated at over £1,000,000. The Medical Superintendent is Major Norcliffe Roberts, O.B.E., M.D.

### The Council's Activities.

The Council in 1889, through the apportionment of County property as between the old geographical counties of Middlesex, Surrey and Kent, had transferred to it the mental hospitals at Hanwell, Colney Hatch, and Cane Hill, and the foundations of a fourth hospital at Claybury. Since that date the Council has completed the superstructure of Claybury Mental Hospital, which was opened in 1893, has provided Bexley Mental Hospital, which was opened in 1898, and purchased in 1896 the Horton Estate, Epsom, upon which the Manor (1899), Horton (1902), The Epileptic Colony (1903), and Long Grove (1907) mental hospitals have been built.

### A SYDENHAM CHILDREN'S HOSPITAL.

On Friday, June 20, the new out-Patient Department of the South Eastern Hospital for Children at Sydenham was opened by Princess Beatrice, who is a Patron of the hospital, in the presence of a large number of visitors. Sir Philip Dawson, M.P., read a short address of welcome to Her Royal Highness and a speech was made by the Mayor of Lewisham, in which he stated that the new department was being opened entirely free of debt. After Princess Beatrice had left the visitors were invited to inspect the new building, which is certainly a most valuable addition to the hospital. On the ground floor there are about a dozen rooms, including operating theatre, dispensary, dental room and X-ray room, and above this is a special floor where cases can be isolated. The building, without any equipment has cost £10,000.

### THE NEW ERA IN ASYLUMS.

### WEST PARK MENTAL HOSPITAL.

THE ADMINISTRATIVE BLOCK. [*Newbery, Brixton.*

A GROUP OF WARDS THE HOSPITAL ESTATE IN THE BACKGROUND. [*Newbery, Brixton.*

(See page 209.)

## Figures and Tables

**Figure f1:**
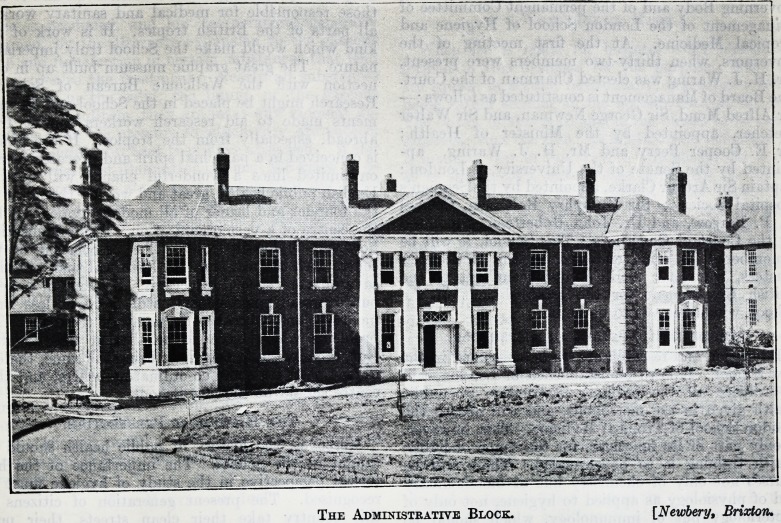


**Figure f2:**